# Fully Automated Segmentation of Cervical Spinal Cord in Sagittal MR Images Using Swin-Unet Architectures

**DOI:** 10.3390/jcm14196994

**Published:** 2025-10-02

**Authors:** Rukiye Polattimur, Emre Dandıl, Mehmet Süleyman Yıldırım, Utku Şenol

**Affiliations:** 1 Department of Electronics and Computer Engineering, Institute of Graduate, Bilecik Şeyh Edebali University, 11230 Bilecik, Türkiye; rukiye.polattimur@bilecik.edu.tr; 2 Department of Computer Engineering, Faculty of Engineering, Bilecik Şeyh Edebali University, 11230 Bilecik, Türkiye; 3 Department of Computer Technology, Söğüt Vocational School, Bilecik Şeyh Edebali University, Sögüt, 11600 Bilecik, Türkiye; mehmets.yildirim@bilecik.edu.tr; 4 Department of Radiology, Medical Faculty, Akdeniz University, 07070 Antalya, Türkiye; utkusenol@akdeniz.edu.tr

**Keywords:** cervical spinal cord segmentation, sagittal MRI, deep learning, U-Net, vision transformers, Swin-Unet

## Abstract

**Background/Objectives**: The spinal cord is a critical component of the central nervous system that transmits neural signals between the brain and the body’s peripheral regions through its nerve roots. Despite being partially protected by the vertebral column, the spinal cord remains highly vulnerable to trauma, tumors, infections, and degenerative or inflammatory disorders. These conditions can disrupt neural conduction, resulting in severe functional impairments, such as paralysis, motor deficits, and sensory loss. Therefore, accurate and comprehensive spinal cord segmentation is essential for characterizing its structural features and evaluating neural integrity. **Methods**: In this study, we propose a fully automated method for segmentation of the cervical spinal cord in sagittal magnetic resonance (MR) images. This method facilitates rapid clinical evaluation and supports early diagnosis. Our approach uses a Swin-Unet architecture, which integrates vision transformer blocks into the U-Net framework. This enables the model to capture both local anatomical details and global contextual information. This design improves the delineation of the thin, curved, low-contrast cervical cord, resulting in more precise and robust segmentation. **Results**: In experimental studies, the proposed Swin-Unet model (SWU1), which uses transformer blocks in the encoder layer, achieved Dice Similarity Coefficient (DSC) and Hausdorff Distance 95 (HD95) scores of 0.9526 and 1.0707 mm, respectively, for cervical spinal cord segmentation. These results confirm that the model can consistently deliver precise, pixel-level delineations that are structurally accurate, which supports its reliability for clinical assessment. **Conclusions**: The attention-enhanced Swin-Unet architecture demonstrated high accuracy in segmenting thin and complex anatomical structures, such as the cervical spinal cord. Its ability to generalize with limited data highlights its potential for integration into clinical workflows to support diagnosis, monitoring, and treatment planning.

## 1. Introduction

The spinal cord is one of the most critical structures of the central nervous system. It serves as a fundamental neural pathway for transmitting signals between the brain and the rest of the body. Extending from the brainstem to the coccyx, the spinal cord is located within an anatomical system consisting of the cervical, thoracic, lumbar, and sacral regions [1,2]. The cervical region of the spinal cord is particularly important for controlling the head and neck and arm movement, as well as managing basic life functions [3]. Damage to the cervical spinal cord can result in severe neurological disorders caused by trauma, tumors, infections, and degenerative diseases [4,5]. Therefore, computerized diagnostic and surgical application tools with fully automated segmentation techniques that accelerate clinical processes are essential [6,7].

Recently, many models have been developed to obtain additional information from routine imaging scans [8]. Thanks to advances in medical imaging techniques and the non-invasive nature of the procedure, it is now possible to perform a comprehensive analysis of the spinal cord without harming the patient. Due to advancements in magnetic resonance (MR) imaging technologies, the structure and pathological changes in the spinal cord can be analyzed with high accuracy. The noninvasive nature, multiplanar imaging capacity, and soft tissue sensitivity of magnetic resonance imaging (MRI) make it an indispensable tool for diagnosing and monitoring neurological diseases, particularly multiple sclerosis, tumors, and traumatic spinal cord injuries [9,10]. Thus, spinal cord segmentation plays a critical role in many areas, including diagnosing neurological diseases, planning surgical interventions, and designing rehabilitation processes [11]. However, the spinal cord’s long, curved, and thin structure makes imaging and segmentation challenging [12]. Traditional segmentation methods, such as manual drawing, edge detection, and region growing, require a high level of expertise. Recently, however, fully automated, deep-learning-based approaches have provided more accurate, faster, and clinically applicable solutions [11,13].

Convolutional neural networks (CNNs), particularly the U-Net architecture and its variants, have become the important architecture for many medical image segmentation tasks due to their encoder–decoder structure and ability to learn multi-scale features [14]. However, CNN-based methods are limited in their ability to capture long-range dependencies and global context due to their localized receptive fields. This limitation becomes particularly evident in spinal cord segmentation tasks, especially in the cervical region, where the cord is thin, curved, and has low contrast against the surrounding tissues. Vision Transformers (ViTs) have recently emerged as a powerful alternative in medical imaging, overcoming these challenges by incorporating self-attention mechanisms that model global contextual relationships more effectively than traditional CNNs [15]. Among these, the Swin Transformer introduces a hierarchical structure with shifted windows that balances computational efficiency with modeling capability. This makes it suitable for dense prediction tasks, such as segmentation [16]. Building upon this advancement, Swin-Unet integrates the hierarchical Swin Transformer blocks into a U-Net-like encoder–decoder architecture. It leverages the benefits of both convolutional feature extraction and global attention mechanisms, enabling the model to capture fine-grained anatomical details and broader structural patterns simultaneously. Swin-Unet has shown state-of-the-art performance in several segmentation benchmarks [17,18,19].

Spinal cord segmentation is critical for diagnosing and monitoring diseases of the central nervous system. It has the potential to inform clinical decisions, especially in cases involving multiple sclerosis (MS), traumatic injuries, tumors, and degenerative diseases. For this reason, previous studies have focused on quantitative analysis-based segmentation approaches. Over time, this field has evolved from traditional image processing methods to deep learning-based models and, most recently, transformer architectures.

Early spinal cord segmentation studies were based on clinical observations and technical investigations. For example, Losseff et al. [9] discovered a significant inverse correlation between spinal cord area and disability level. Thus, the clinical value of spinal cord segmentation was clearly demonstrated. The study provided important evidence that spinal cord volume can be used as a biomarker to assess the progression of neurological diseases, motivating further research. Subsequent semi-automated segmentation studies have examined the relationship between cervical spinal cord volume and disability level using intensity-based [9,20,21], surface-based [22,23,24], and image-based [25,26,27] approaches. These studies have shown that morphological measurements can significantly contribute to clinical follow-up processes.

In spinal cord segmentation, traditional methods have predominantly relied on intensity-based, contour modeling, or atlas-guided strategies, as well as clinically based approaches. Techniques such as active contour models [28], deformable models [29], and graph cuts [27] have provided segmentation based on edge and shape information. These techniques enable the separation of spinal cord structures in regions with limited contrast. However, these methods have shown limited success when confronted with low contrast, motion artifacts, and individual anatomical variations. Consequently, machine learning-based methods supported by predefined gray-level, edge, and texture features have become widespread. In particular, models developed by Gros et al. [30] for spinal cord segmentation demonstrated the potential of these methods for gray and white matter separation. Around the same time, De Leener et al. [31] developed the spinal cord toolbox, provides user-friendly segmentation solutions with atlas-based alignment and semi-automatic slice extraction features.

Although traditional and semi-automated approaches have made significant progress in spinal cord segmentation, a new era has begun with the use of convolutional neural networks (CNNs). The parallel computing capability of graphics processing units (GPUs) has greatly improved the accuracy and speed of segmentation in deep learning-based models. In one of the earliest CNN applications, Perone et al. [32] performed spinal cord and gray matter segmentation using 2D CNN structures. The architecture was later integrated into the spinal cord toolbox and applied to practical uses. The TractSeg model, developed by Wasserthal et al. [33], showed high performance in different MRI modalities and improved clinical compatibility. Three-dimensional (3D) CNN-based models developed by Huo et al. [34] offer the advantage of better spatial context learning. Similarly, deep models trained on multimodal MRI data by Zhu et al. [35] enabled more detailed segmentation while preserving structural integrity. Bédard et al. [36] provided an advancement using a U-Net model toward robust, contrast-independent spinal cord segmentation by leveraging soft-label regression and building a model to unify multiple MRI contrasts. Its integration into the Spinal Cord Toolbox [37] enhanced its practical utility and encourages wider adoption. Karthik et al. [38] introduced SCIseg, a deep learning-based framework for automatic segmentation of the spinal cord and intramedullary lesions in patients with spinal cord injury, using T2-w MRI scans. In another study, Karthik et al. [39] presented a framework that tracks the stability of morphometric measurements derived from automatic spinal cord segmentation models. However, these convolutional structures have limited in their ability to model long-range contextual relationships because they focus primarily on local attributes [40,41]. For longitudinal anatomical structures, such as the spinal cord, this can negatively impact segmentation accuracy and consistency. Transformer-based approaches, which were developed to overcome these shortcomings, have rapidly gained prominence in segmentation thanks to their global context learning capabilities [42].

Vaswani et al. [43] first proposed the transformer architecture, which was then adapted for image processing by Dosovitskiy et al. [15] with the Vision Transformer (ViT) structure. Chen et al. [44] subsequently applied this architecture to medical image segmentation using the TransUNet model. The TransUNet model learns both local and global contextual relationships simultaneously by combining CNN-based encoder layers and transformer blocks. To overcome the TransUNet’s limitations as a hybrid model combining CNN-based encoders and ViT blocks, Cao et al. [17] developed a pure transformer-based the Swin-UNet architecture. Swin-Unet uses only swin transformer blocks to enable multi-scale and hierarchical feature learning, eliminating the need for CNN components and promising more efficient and flexible segmentation performance, especially in complex texture discriminations.

Although several transformer-based models have been applied to spinal or lumbar datasets, applications specifically targeting cervical spinal cord segmentation using Swin-Unet remain extremely limited. Zhang et al. [45] proposed SeUneter, a channel-attentive U-Net variant designed for instance segmentation of cervical spine structures in T2-w MRI slices. The method employed was U-Net with channel-attention modules and performance was compared with TransUNet and Swin-Unet. The study achieved higher segmentation accuracy for cervical spinal cord and canal than baseline models on T2-w cervical MRI images. In another study, Karthik et al. [46] presented a comparative analysis of models capable of consistent spinal cord segmentation across different MRI contrasts. The study evaluated the contrast independence levels of deep learning-based models used in spinal cord cross-sectional area (CSA) measurement. Seven models with different architectures, including CNN, ConvNeXt, non-hierarchical ViT, and hierarchical ViT architectures, were tested on a publicly available dataset. The results showed that hierarchical ViT architectures were more stable than pure ViTs, while pure ViT models were more sensitive to changes in contrast. Consequently, the application of Swin-Unet in spinal cord segmentation is rare in the previous studies. Its effectiveness on sagittal MR images of the cervical spinal cord has not been thoroughly investigated, despite the unique challenges posed by its thin structure, curvature, and low tissue contrast. Therefore, our study addresses this gap by exploring the performance of Swin-Unet on this specific modality and anatomical region. These comprehensive studies evaluate methodological and technological advances in spinal cord segmentation from past to present in relation to clinical needs. The studies presented demonstrate the evolution of segmentation algorithms and their contribution to clinical decision support systems. They also highlight the limitations of applying these algorithms, particularly in structurally complex regions such as the cervical spinal cord.

In this study, we propose a fully automated segmentation framework for the cervical spinal cord in sagittal T2-weighted (T2-w) MR images. Our framework uses the Swin-Unet architecture and aims to enhance segmentation accuracy and consistency, especially in challenging anatomical regions. It also enables rapid clinical assessment and supports early diagnosis. To the best of our knowledge, this is one of the first studies to apply Swin-Unet to the segmentation of cervical spinal cord structures in sagittal MR images with such key performance metrics. The main contributions of this study are outlined as follows:

1. This study presents one of the first applications of the Swin-Unet architecture specifically used for cervical spinal cord segmentation, demonstrating its suitability for modeling the thin, curved, and anatomically complex structures of the spinal cord.
2. The segmentation framework leverages sagittal T2-w MR images, a modality widely used in spinal imaging, ensuring that the proposed method is directly aligned with clinical practice and capable of capturing diagnostically relevant anatomical details.
3. A fully automated and high-accuracy segmentation approach is proposed to eliminate the need for manual annotation and enable faster clinical assessment.
4. The study is supported by an original dataset that provides novel insights and practical relevance for evaluating the proposed model under real clinical conditions.
5. Extensive comparative analyses are performed against baseline and state-of-the-art segmentation methods, demonstrating the superiority of the Swin-Unet-based approach in terms of both pixel-wise accuracy and structural consistency.
6. The clinical applicability of the method is emphasized by evaluating its potential integration into diagnostic and treatment planning workflows, particularly for early detection and follow-up of spinal cord-related pathologies.

This study proposes a fully automated Swin-Unet-based framework to address the challenges of accurately segmenting the cervical spinal cord, particularly its thin, curved, and low-contrast structure in sagittal T2-w MR images. The model captures both fine-grained anatomical details and global structural patterns by integrating hierarchical attention mechanisms, thereby improving segmentation accuracy and consistency. The study focuses on developing a clinically applicable tool that supports the rapid assessment and early diagnosis of spinal cord-related pathologies, as well as follow-up. Through extensive evaluation on an original dataset, the framework aims to demonstrate superior performance compared to existing methods, bridging the gap between advanced computational techniques and practical clinical needs. The proposed method’s framework is open-source, and the code for segmentation tasks can be found in the GitHub platform [47].

## 2. Materials and Methods

**Figure 1:**
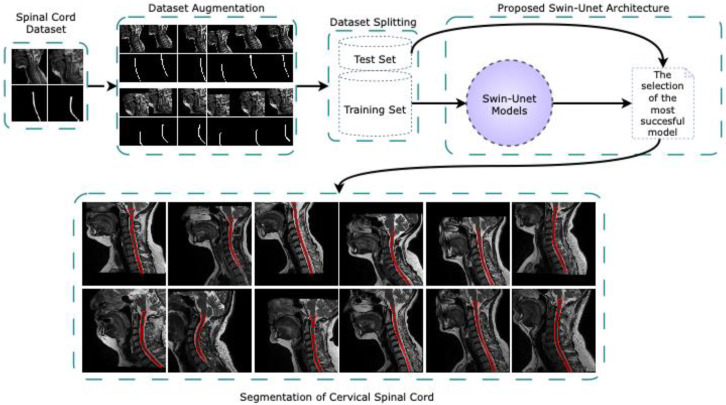
The overall pipeline of the proposed fully automated cervical spinal cord segmentation framework based on Swin-Unet architectures.

This study performs fully automated segmentation of the CSA in the cervical spinal cord is performed using Swin-Unet architectures on sagittal MR images in a new dataset created for the cervical spinal cord. Figure 1 delineates the comprehensive pipeline of the proposed fully automated cervical spinal cord segmentation framework based on Swin-Unet architectures. The workflow is structured into five primary stages such as spinal cord dataset acquisition, dataset augmentation, dataset splitting, segmentation via Swin-Unet models, and visualization of the segmentation results. The initial input to the system consists of sagittal T2-weighted MR images containing the cervical spinal cord. These images form the basis for the subsequent preprocessing and model training. The dataset is curated to ensure clinical relevance and anatomical diversity for robust model generalization. In dataset augmentation stage, various augmentation techniques such as horizontal and vertical shift, rotation and zoom are applied to artificially expand the dataset. This process enhances the model’s ability to generalize to unseen variations in spinal cord morphology and image acquisition conditions. The augmented images maintain anatomical fidelity while introducing the diversity that is essential for preventing overfitting. The augmented dataset is partitioned into training and test sets using stratified sampling to preserve class distribution. The training set is used for model optimization, while the test set is reserved for performance evaluation, ensuring unbiased assessment of segmentation performance. Several Swin-Unet models are trained on the training dataset. Swin-Unet, a hybrid architecture that integrates the swin transformer with the traditional U-Net, leverages both global context modeling and spatial localization capabilities. Different configurations of Swin-Unet are evaluated, and the model achieving the best performance on the validation subset is selected for final evaluation. The selected Swin-Unet model is deployed to perform segmentation on the cervical spinal cord in the sagittal MR images from the test set. The segmented outputs with highlighted in red contours are visualized alongside their corresponding ground truth images. The figure demonstrates consistent and accurate delineation of the spinal cord boundaries across different samples, highlighting the effectiveness of the proposed method.

### 2.1. Dataset

In this study, an original dataset for automatic segmentation of the cervical spinal cord CSA region was created from T2-w sagittal MR images. MR scans of the patient group were performed using a 3.0 T Siemens Magnetom Spectra MR scanner at Akdeniz University Hospital. A total of 98 patients were included in the dataset, 23 of whom were male and 75 of whom were female. The patients ranged in age from 18 to 72. Data collection was conducted over a period of two years, concluding in May 2024. In addition, the conduct of the study and the preparation of the dataset were approved by the decision of the Clinical Research Ethics Committee of Faculty of Medicine of Akdeniz University on 15 September 2021, under the number KAEK-644.

The MR images in the dataset were acquired with a matrix size of 384 × 384 pixels. Depending on the patient’s anatomy, this resulted in 13 to 15 slices per scan. The original dimensions of the MR images were preserved, and they were not subjected to preprocessing, such as cropping or resizing. This approach is important for maintaining data integrity in medical imaging studies because changes in image dimensions can affect segmentation accuracy [48]. The slice thickness in the MRIs was set to 3 mm, and the gap between slices was set to 3.3 mm. NEX = 3.00 was used to reduce noise in the images. The voxel dimensions were 0.78125 × 0.78125 × 3.3 mm^3^, corresponding to a voxel volume of approximately 2.013 mm^3^, to achieve high anatomical detail. The imaging sequence used was a sagittal T2-w turbo spin echo (T2_TSE_SAG) protocol in 2D acquisition mode. The basic scan parameters were set as follows: repetition time (TR) = 3000 ms; echo time (TE) = 97 ms; and flip angle = 15°. The radiofrequency (RF) signal frequency used during imaging was 123.185655 Hz; the pixel bandwidth was 260 Hz; the partial sampling rate was 70%; and the image field phase ratio was 100%.

For segmentation, CSA of the spinal cord corresponding to each image in the dataset prepared for the study was manually labeled by experts using ITK-SNAP 4.0 software [49], and ground truth (GT) masks were created. The main criteria for creating GT masks were image quality and the cervical region. Age, gender, and clinical diagnosis were not considered. All scans in the dataset were provided in DICOM (Digital Imaging and Communications in Medicine) format, which is widely used in digital medical imaging. After analyzing and sorting the sagittal MR images of the cervical spinal cord, they were converted to NIfTI (Neuroimaging Informatics Technology Initiative) format, which is leaner and widely supported by neuroimaging software ITK-SNAP. ITK-SNAP software was used for this conversion, and parameters such as voxel size and spatial resolution were preserved. As a result, 217 suitable MR slices from the patient MR scans in the dataset were selected for cervical spinal cord segmentation.

**Figure 2:**
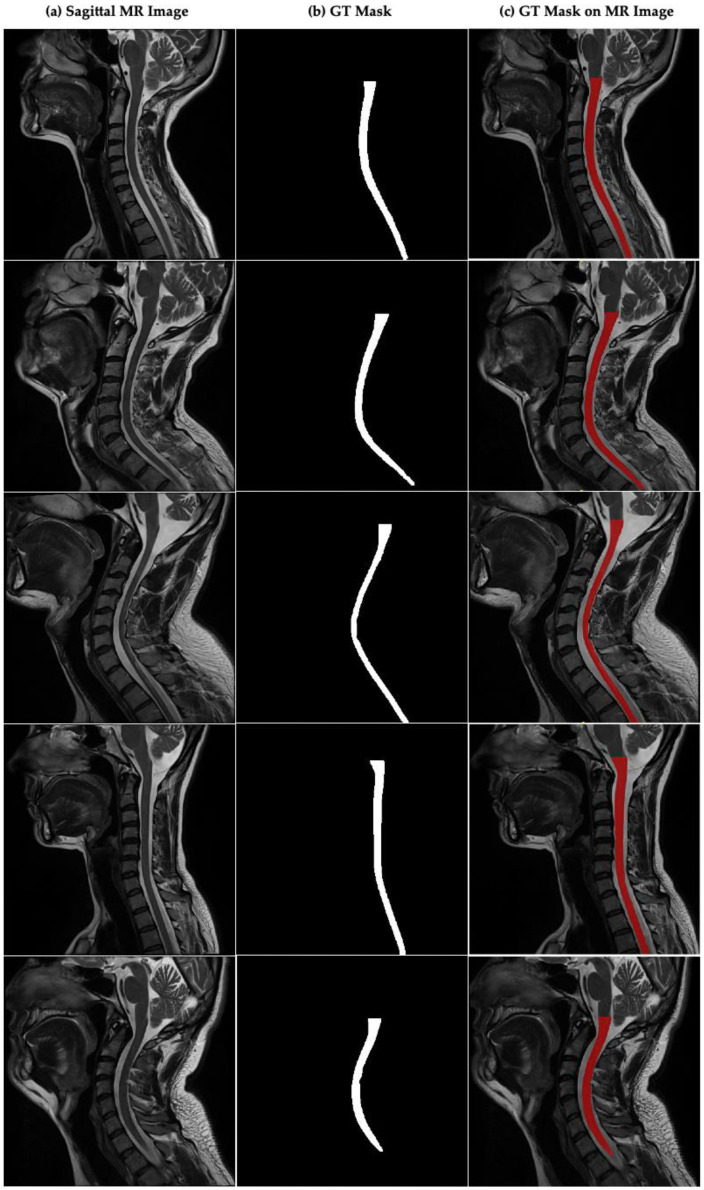
Sagittal plane MR image of the cervical spinal cord and CSA boundaries annotated by the experts in cervical spinal cord. (**a**) Sagittal MR images in dataset, (**b**) GT mask annotated by experts for cervical spinal cord, (**c**) GT mask of CSA on sagittal MR image.

Figure 2a–c presents sample sagittal MR images of the cervical spinal region, the corresponding ground truth (GT) spinal cord segmentation masks that were manually annotated by expert radiologists, and the overlay visualizations of these masks on the original MR images. The image shows the expert-annotated ground truth masks for each image and illustrates the overlay of these masks on their respective MR images for visual validation. The masks are highlighted in red to clearly indicate the location and boundaries of the spinal cord.

### 2.2. Data Augmentation

The creation of medical image datasets is a very challenging process due to ethical concerns, the high cost of data collection, and various other factors. It is considered the biggest limiting factor in studies [50]. Often, it is not possible to obtain a sufficient number of samples by working using raw data alone. Data augmentation techniques can contribute positively to the training process by expanding the existing dataset. Data augmentation is a method that uses certain algorithms to expand the training data and reduce the negative effects caused by image distortions [51].

This study applies data augmentation methods based on geometric transformations to improve the segmentation performance of the proposed Swin-Unet models and stabilize the learning process. Despite the limited number of images in the dataset, spinal cord CSA is considered a factor that increases the efficiency of the data augmentation process due to its unique shape. During the data augmentation phase, we applied horizontal and vertical shifting, rotation, and zooming techniques to create artificial samples. Specifically, the image set was expanded by applying rotation, shift (in the horizontal and vertical axes), and zoom operations without disturbing the pixel structure. It is critical to apply the same transformations to the image and the mask during these operations, as a meticulous and attentive approach is required at this stage; this directly affects model performance. As a result of the data augmentation process carried out within this framework, the number of image-mask pairs increased from 217 to 869.

### 2.3. The Proposed Methodology

In this study, transformer and U-Net based Swin-Unet deep learning architectures are used for fully automated segmentation of the cervical spinal cord from sagittal T2-w MR images. This segmentation method takes advantage of Swin-Unet’s ability to model long-range contextual relationships, including those of the cervical spinal cord.

#### 2.3.1. Swin-Unet

In recent years, there have been rapid developments in deep learning-based image processing methods, particularly transformer architectures. The transformer architecture was first proposed by Vaswani et al. [43] and works with only a self-attention mechanism, eliminating the need for sequential structures, such as recurrent neural networks. The vision transformer (ViT) architecture, inspired by phrases and proposed by Dosovitskiy et al. [15], processes images into fixed-size chunks and has achieved competitive results in visual tasks such as medical image analysis and segmentation. Transformer structures are a more effective alternative to the limited field-of-view-based feature extraction of traditional CNN architectures, especially due to their ability to model long-range dependencies and attention. While CNNs are successful in local feature extraction, they struggle to model long-range contextual relationships. This leads to performance loss, particularly in medical images, where structural integrity must be preserved and contextual information must be modeled accurately.

In tasks where spatial context is critical, such as image segmentation, transformer architectures offer high performance and generalizability by evaluating the entire image simultaneously using global attention mechanisms. Synthesis architectures, which combine CNNs local information extraction with transformers’ contextual awareness, have emerged as a promising solution. TransUNet, one of the synthesis architectures proposed by Chen et al. [44], combines convolution layers and transformers based on the attention mechanism. By integrating transformer modules into the encoder, decoder, and skip connection components of the classical U-Net architecture [14], TransUNet can learn both local and global contextual information simultaneously.

In segmentation, the global attention mechanism cannot directly capture local features, so additional methods are needed to model such information. One method is integrating pre-trained convolutional architectures, such as ResNet, EfficientNet, or convNet, into transformer architectures to extract local features. This enables the efficient learning of details and overall structure through local, window-based, self-attention mechanisms that focus on small regions of the target workspace, as well as multi-scale feature extraction techniques. The swin transformer architecture effectively uses the attention mechanism by implementing small, local, window-based blocks and shifted windows to transfer information between different windows [16]. This method significantly reduces computational costs and enables the multi-level learning of local and global contexts within a hierarchical structure. Thus, the swin transformer structure achieves more successful segmentation results in structurally complex and challenging images, such as those of the spinal cord, than the traditional transformer structure does.

The Swin-Unet architecture, proposed by Cao et al. [17], is a deep learning architecture characterized by a transformer-based encoder–decoder structure integrated into the U-Net architecture. This structure has strong structural features. The swin transformer blocks in the encoder part of the Swin-Unet architecture enable hierarchical and multi-scale feature extraction using small window-based self-attention. The shifted window attention mechanism in these blocks allows the model to capture local and global contextual relationships by exchanging information between windows. In the decoder, this multi-level representation is transformed into high-resolution, semantically consistent segmentation masks via patch expansion layers and reconstruction steps. Thanks to its fully transformer-based architecture and hierarchical attention mechanisms, the Swin-Unet architecture provides a significant advantage in segmentation performance, enabling more powerful feature extraction and more efficient learning of long-range contextual relations [17].

Swin-Unet addresses the challenges commonly encountered in medical imaging, such as limited resolution, low contrast, and similar tissue structures. It offers the capacity to perform detailed feature extraction and representation learning in a large context thanks to its multi-scale, window-based attention mechanisms. In regions with thin, long, and complex anatomical structures, such as the cervical spinal cord, this architecture’s careful, multi-level information processing capability strengthens segmentation accuracy and consistency. Furthermore, Swin-Unet’s fully transformer-based structure enables the model to effectively model large contextual relationships and adapt to anatomical variations in different individuals. In this respect, Swin-Unet is a powerful, flexible solution that stands out among current deep learning approaches, particularly for achieving high performance with limited data.

#### 2.3.2. The Proposed Swin-Unet Architectures

**Figure 3:**
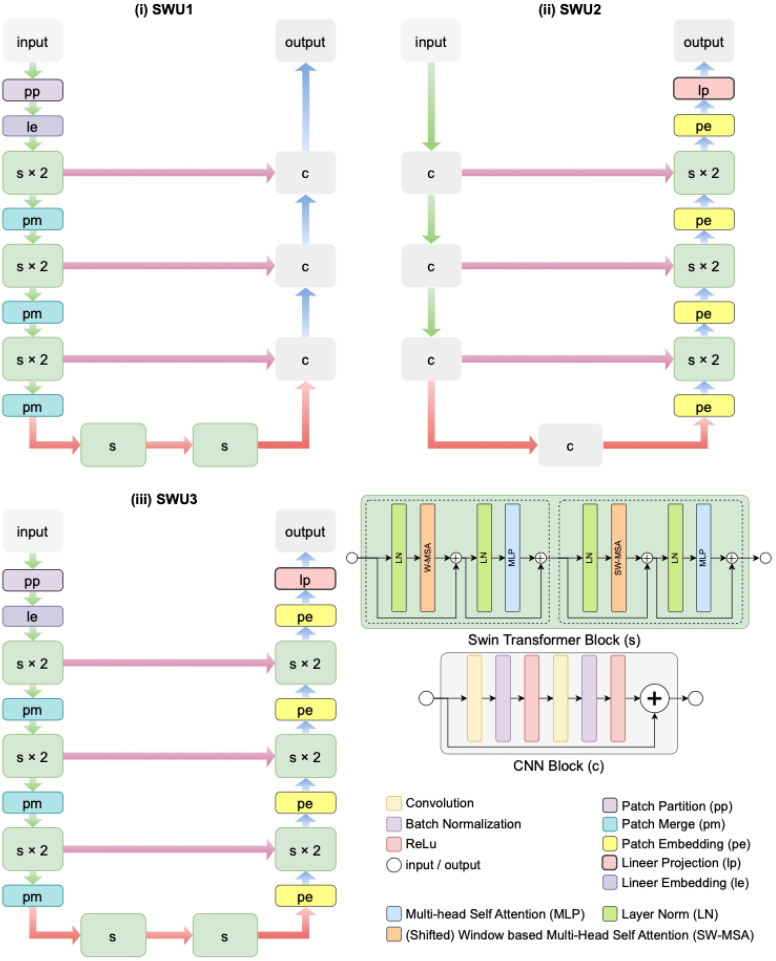
The architectural schematics three distinct variations in the Swin-Unet architectures proposed for the fully automated segmentation of the cervical spinal cord in sagittal MR images.

Different variants can be created in image segmentation by using swin transformer blocks in the encoder, decoder, and both encoder and decoder parts of the Swin-Unet architecture [52]. In this study, three distinct variations in the Swin-Unet architecture-namely SWU1, SWU2, and SWU3-have been proposed and comparatively evaluated for the fully automated segmentation of the cervical spinal cord in sagittal MR images. Each architecture leverages the strengths of swin transformer blocks and CNNs, with a strategic variation in the location of these components within the encoder–decoder framework to assess their impact on segmentation performance. The architectural schematics of these models are illustrated in Figure 3.

In SWU1, as seen in Figure 3i, swin transformer blocks (denoted as s) are exclusively incorporated into the encoder part of the architecture. The input image undergoes patch partitioning (pp), linear embedding (le), and two stages of swin transformer blocks (each repeated twice), followed by patch merging (pm). This hierarchical feature extraction pipeline allows for capturing long-range dependencies in the input through window-based self-attention mechanisms. The decoder consists of standard CNN blocks (denoted as c), which reconstruct the segmentation map via successive upsampling and convolutional layers. Skip connections are used to transfer feature maps from the encoder to the decoder at corresponding resolutions, enabling spatial detail preservation. In contrast, SWU2 in Figure 3ii deploys swin transformer blocks only in the decoder. The encoder is built entirely with CNN blocks, which extract local spatial features through convolutional operations. The decoder integrates swin transformer blocks (s × 2) and patch embedding (pe) modules to exploit global contextual information during reconstruction. Linear projection (lp) and patch merging (pm) steps are also utilized in the decoder path. This design investigates the influence of transformer-based decoding on improving the refinement and boundary accuracy of segmentation results.

SWU3 in Figure 3iii represents a hybrid architecture that embeds swin transformer blocks in both the encoder and decoder paths. The encoder initiates with patch partitioning and linear embedding, followed by hierarchical Swin Transformer stages (s × 2), interleaved with patch merging. The decoder mirrors this structure with patch embedding and additional swin transformer stages, ensuring both global and local information is effectively captured and preserved throughout the network. This full integration of transformer blocks enables the model to learn rich feature representations while maintaining structural integrity via skip connections and layer normalization.

## 3. Experimental Results

In this study, cervical spinal cord segmentation was performed with Swin-UNet, one of the transformer-based U-Net architectures, using sagittal T2-w MR images in the prepared dataset. All experiments were conducted on a computer system equipped with an Intel Core i5 4.10 GHz processor, 16 GB of DDR4 3000 MHz RAM, an NVIDIA RTX A4000 16 GB GPU, a 1 TB HDD, and a 500 GB SSD. MRI image analysis, manual segmentation, and generation of the ground truth (GT) mask were performed using ITK-SNAP 4.0 software. Model development, training, and evaluation were carried out in the Jupyter Notebook environment (v. 7.1.2) using Python language (v. 3.6.13) and the PyTorch (v. 2.0.0) deep learning library. Additionally, the Timm (PyTorch Image Models) library was used for ViT and other convolutional models. We trained and validated the segmentation models using cervical spinal cord MR images and the corresponding ground truth (GT) masks manually generated from the dataset.

Since the spinal cord could not be clearly visualized in every slice of the MR images in the dataset, experts selected anatomically appropriate slices and applied manual masking to them. A total of 217 MR images and corresponding masks were created using data from 98 patients. For the study, the images were divided into two subsets: 80% for training and 20% for testing and validation, to prevent overfitting during model evaluation. Using data augmentation, 869 augmented MR images and expert mask images were obtained for the training set (691 images) and the test set (178 images). All data augmentation procedures were performed in Python using the NumPy library (v. 1.19.5). The following parameters were used for data augmentation: width_shift_range = 0.20, height_shift_range = 0.20, zoom_range = 0.05, and rotation_range = 2.

Certain hyperparameters and training strategies were applied during the training of the proposed SWU1, SWU2, and SWU3 Swin-Unet architectures. During training, the batch size was set to 8 and the number of 384 × 384 images to be processed in each iteration was set to 8. The total number of epochs for training the models was set to 100, and the learning rate was set to 1 × 10^−4^ for optimization. Training was performed on a CUDA-based GPU depending on hardware support. The ComboLoss function, which is an equal-weighted combination of binary cross-entropy and Dice loss (bce_weight = 0.5, dice_weight = 0.5), was chosen as the loss function for the Swin-Unet architecture used for segmentation. The Adam optimization algorithm was used to update the models’ weights. Conversely, the model outputs were normalized using sigmoid activation and converted into a binary mask with a threshold of 0.5.

The Swin-Unet architecture uses a swin transformer-based encoder or decoder structure. Similarly, the encoder and decoder parts of the architecture use a U-Net-like deep learning structure. Thus, Swin-Unet combines the power of the swin transformer to model global context with the capacity of the U-Net to preserve local details, providing highly accurate, structurally consistent segmentation results. In this study, three different Swin-Unet-based architectures-SWU1, SWU2, and SWU3-were designed and evaluated for the task of cervical spinal cord segmentation on sagittal MR images. The placement of swin transformer blocks within the encoder and decoder modules was altered for each architecture to assess the impact of global attention mechanisms on segmentation performance.

The SWU1 model employs a swin transformer backbone exclusively in the encoder, using the pretrained swin_base_patch4_window12_384 architecture. This backbone extracts hierarchical features at four different resolution levels, capturing rich global contextual representations of the spinal cord structure. The decoder in SWU1 follows a conventional CNN-based structure composed of bilinear upsampling, skip connections, and convolutional layers with batch normalization and ReLU activations. This configuration enables efficient spatial reconstruction while maintaining a relatively lightweight structure. SWU1 combines the advantages of transformer-based global feature extraction and convolutional detail recovery, providing a balanced approach suitable for most clinical scenarios. In contrast, the SWU2 architecture incorporates a purely convolutional encoder, consisting of sequential convolutional blocks with downsampling via stride-2 convolutions. The decoder integrates swin transformer blocks at each upsampling stage. This design allows the model to focus on extracting local features in the encoding phase while enhancing contextual awareness during decoding. The attention-based decoding process improves the refinement of boundary regions, which is particularly advantageous in segmenting narrow anatomical structures like the cervical spinal cord. The most comprehensive architecture, SWU3, integrates swin transformer blocks in both the encoder and decoder. Like SWU1, it uses a hierarchical swin transformer encoder to extract multi-scale features. However, unlike the previous models, it also applies swin decoder blocks during reconstruction, ensuring that both local and global dependencies are modeled throughout the network.

The Swin-Unet models (SWU1, SWU2, and SWU3) proposed in this study varied in their performance when different pre-trained, deep-learning-based encoder structures (TU1-TU12 and DTU) of the TransUNet architecture were used. Their performance was then compared using a cervical spinal cord segmentation dataset. Given the positive effects of these studies on segmentation performance, we evaluated the pre-trained transformer-based architectures in Table 1, especially for spinal cord segmentation. The spinal cord is thin, long, and structurally complex. The pretrained architectures used include the classic ResNet series (ResNet-34, ResNet-50, ResNet-101, and ResNet-152), advanced variants of these architectures (ResNet-50d, ResNet-101d, ResNeXt50, ResNeSt50d, and ResNetV2); and typical CNN architectures (EfficientNet, ConvNeXt-Tiny, and RegNet-Y_040). Additionally, we investigated the contributions of the structure integrated in the decoder block (Deco-TransUNet) and the decoder transformer structure to cervical spinal segmentation performance. This diversity was important for evaluating the impact of models with different depths, contextual learning abilities, and parameter efficiencies on segmentation performance. In this context, the SWU1, SWU2, and SWU3 models, created by integrating the swin transformer into the U-Net architecture in various ways, were analyzed from start to finish for their contribution to spinal cord segmentation. In conclusion, the Swin-UNet architecture is recommended for spinal cord segmentation tasks due to its superior accuracy and structural integrity preservation.

**Figure 4:**
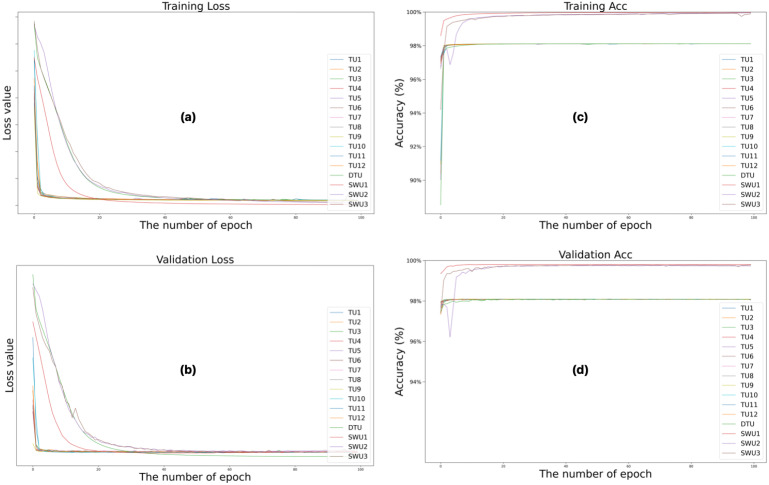
Changes in loss and accuracy values of the training and validation phases of the training processes of Swin-Unet and TransUNet deep learning models during 100 epochs. (**a**) training loss, (**b**) validation loss, (**c**) training accuracy and (**d**) validation accuracy.

Figure 4 illustrates the changes in loss and accuracy values during the training and validation phases to quantitatively evaluate the training processes of the Swin-Unet and TransUNet deep learning models over 100 epochs. Figure 4a shows the training loss, Figure 4b shows the validation loss, Figure 4c shows the training accuracy, and Figure 4d shows the validation accuracy. These figures show the results of training the architectures. Analyzing the loss and accuracy values of the training and validation processes reveals that the Swin-Unet (SWU1, SWU2, and SWU3) architectures yield more successful results than the TransUNet (TU1–TU12 and DTU) variants. While all models exhibit a general downward trend in training loss over time, Swin-Unet-based models achieve lower loss values earlier and demonstrate a more stable learning process. The training accuracy data shows that Swin-Unet architectures stand out with accuracy rates above 99%, while some TransUNet models remain in the 96–98% range. Similarly, validation loss and accuracy metrics show that Swin-Unet architectures have lower validation loss and higher validation accuracy, indicating greater generalizability. These results suggest that Swin-Unet architectures perform more effectively and consistently in cervical spinal cord segmentation, particularly when data is limited.

Obtaining GT masks of sagittal MR images for segmentation of the cervical spinal cord cross-sectional area is critical because it directly affects the segmentation metric results. Therefore, correct labeling at this stage directly correlates with the results obtained. This study evaluates the experimental results of the proposed Swin-Unet architectures for cervical spinal cord CSA segmentation end-to-end using key metrics based on distance, volume, and pixel matching. The first key metric is the Dice Similarity Coefficient (DSC) in Equation (1), which is a similarity-based metric that calculates the ratio of pixel overlap between the ground truth mask (GTM) determined by an expert and the predicted mask (PRM) by the proposed method [53,54].

Volume Overlap Error (VOE) is a metric used for volume-based evaluations. In Equation (2), VOE is a performance metric that represents errors in segmentation overlap [55]. Hausdorff Distance 95 (HD95) in Equation (3), on the other hand, is used for distance-based geometric evaluation of segmentation performance. HD in Equations (4) and (5) calculates the maximum point-wise distance between the ground truth (GT) segmentation and the predicted segmentation in cervical spinal cord segmentation. HD95 improves upon the measurement calculated with HD by taking the 95th percentile into account and reducing the effect of outliers [54]. Recall (REC) in Equation (6) and precision (PRE) in Equation (7) were used to evaluate pixel detection performance in cervical spinal cord segmentation. True positive (TP), false negative (FN), and false positive (FP) values come from the confusion matrix.

(1) $$DSC\;(PRM,GTM)=\frac{2\left|PRM\cap GTM\right|}{\left|PRM\right|\cup \left|GTM\right|}$$

(2) $$VOE\left(PRM,GTM\right)=(1-\frac{\left|PRM\cap GTM\right|}{\left|PRM\right|+\left|GTM\right|-\left|PRM\cup GTM\right|})$$

(3) $$HD95\left(PRM,GTM\right)=\max\left(HD(PRM,GTM),HD(GTM,PRM\right))$$

(4) $$HD\;(PRM,GTM)=\underset{x\in \mathrm{PRM}}{\max}\underset{y\in \mathrm{GTM}}{\min}{\left\|x-y\right\|}_{2}$$

(5) $$HD\;(GTM,PRM)=\underset{y\in \mathrm{GTM}}{\max}\underset{x\in \mathrm{PRM}}{\min}{\left\|x-y\right\|}_{2}$$

(6) $$REC\;(PRM,GTM)=\frac{TP}{TP+FN}$$

(7) $$PRE\left(PRM,GTM\right)=\frac{TP}{TP+FP}$$

The results of TransUNet models with different pre-trained convolutional architectures and Swin-UNet architectures were comparatively evaluated for cervical spinal cord segmentation in the experimental studies carried out within the scope of the study. Table 2 presents a comprehensive quantitative evaluation of the proposed Swin-Unet-based models-SWU1, SWU2, and SWU3-alongside twelve pretrained convolutional backbone-based TransUNet variants (TU1–TU12) and a default decoder-based TransUNet configuration (DTU) for the task of segmenting the cross-sectional area of the cervical spinal cord from sagittal MR images. Performance is assessed using five widely accepted segmentation metrics such as DSC, PRE, REC, HD95, and VOE. Among all evaluated models, SWU1 achieved the highest overall performance, with a DSC of 0.9526, PRE of 0.9477, and REC of 0.9587, indicating both high boundary adherence and volumetric overlap with the ground truth. Furthermore, SWU1 also achieved the lowest HD95 (1.0707 mm) and lowest VOE (0.0896) among all models, highlighting its superior boundary accuracy and spatial precision. These results reflect the effectiveness of utilizing Swin Transformer blocks in the encoder combined with a CNN-based decoder, which leverages global context for feature extraction and efficient local reconstruction. SWU3, which employs swin transformer blocks in both encoder and decoder, also delivered strong performance across all metrics. It reached a DSC of 0.9456, PRE of 0.9473, and REC of 0.9458, demonstrating balanced sensitivity and specificity. Although SWU3’s boundary accuracy (HD95 = 1.1235 mm) is slightly inferior to SWU1, it still outperforms all TransUNet models in this regard, confirming the added benefit of transformer-based attention mechanisms on both sides of the network. In contrast, SWU2, which features a CNN-based encoder and a transformer-based decoder, showed comparatively lower performance (DSC = 0.9365, PRE = 0.9287, REC = 0.9467) and exhibited a significantly higher boundary error (HD95 = 1.6959 mm) and VOE (0.1183). These results suggest that placing the Swin Transformer only in the decoder may limit its ability to capture global semantic features early in the representation hierarchy, thus reducing its overall effectiveness.

When compared to the pretrained TransUNet models, the SWU variants, especially SWU1 and SWU3, consistently outperformed all TransUNet models across nearly all evaluation metrics. For instance, although some TransUNet models such as TU3 and TU10 showed respectable performance (TU3 with DSC = 0.9408 and HD95 = 0.9306 mm), they were unable to match the combined performance of Swin-based architectures, particularly in terms of volumetric overlap and prediction consistency. In conclusion, the results clearly demonstrate that incorporating swin transformer blocks, particularly in the encoder (SWU1) or both encoder and decoder (SWU3), substantially enhances the model’s capacity to accurately segment complex anatomical structures such as the cervical spinal cord. This improvement is especially evident in the boundary-focused metrics (HD95 and VOE), which are critical for clinical reliability. On the other hand, taking into account DSC scores, the Wilcoxon two-sided rank test was used to determine the statistical significance of the architectures’ results in Table 2 for cervical spinal cord segmentation from sagittal MR images. When SWU1 and the other architectures were evaluated statistically according to the Wilcoxon two-sided rank test, a *p*-value of 0.0006 (*p* = 0.0006) was obtained. Since *p* < 0.05, the results obtained with SWU1 were considered statistically significant. Furthermore, when SWU2 and SWU3 were evaluated against the other architectures using the Wilcoxon test, *p*-values of 0.0021 (*p* = 0.0021) and 0.0045 (*p* = 0.0045) were obtained, respectively. Therefore, as *p* < 0.05, the results obtained with SWU2 and SWU3 were also considered statistically significant according to the Wilcoxon two-sided rank test.

**Figure 5:**
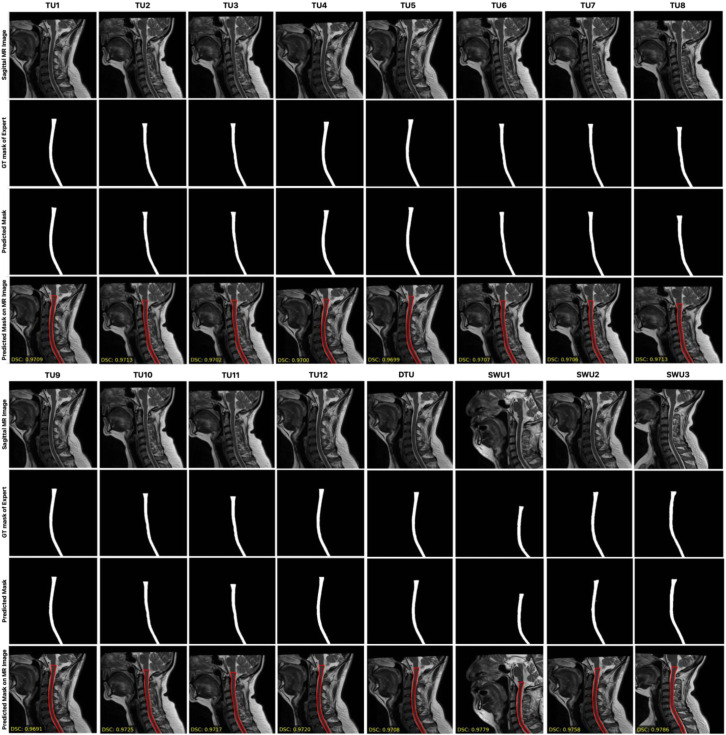
The comparison of the segmentation performance across multiple models, including TransUNet variants and the three proposed Swin-Unet-based models with DSC scores.

Figure 5 presents a qualitative comparison of the segmentation performance across multiple models, including TransUNet variants and the three proposed Swin-Unet-based models (SWU1, SWU2, SWU3) with DSC scores. For each model, it is shown the original sagittal MR image, the corresponding ground truth (GT) segmentation mask, and the predicted segmentation result overlaid on the MR image. This layout enables direct visual evaluation of the alignment between model predictions and expert-annotated GT across anatomically varying cervical spinal cord regions. Upon visual inspection, the SWU1 model demonstrates the closest visual agreement with the GT, successfully capturing the thin, elongated structure of the cervical spinal cord and maintaining consistent boundary alignment across the entire region of interest. Its contours are smooth, precise, and well-aligned with the true anatomical boundary, suggesting a strong ability to model both global and local features. SWU3, which incorporates swin transformer blocks in both the encoder and decoder, also shows high-fidelity segmentation. In contrast, SWU2, which uses CNNs in the encoder and swin transformer blocks in the decoder, shows less accurate results in comparison to SWU1 and SWU3. The results from TransUNet models exhibit varying degrees of segmentation performance. Overall, this qualitative analysis visually confirms that the Swin-Unet variants, particularly SWU1 and SWU3, outperform standard TransUNet-based architectures in preserving the anatomical integrity of the spinal cord in sagittal MR images. Their ability to leverage transformer-based attention mechanisms contributes to more accurate, reliable, and clinically usable segmentation outcomes.

## 4. Discussion

Examining previous studies on cervical spinal cord cross-sectional segmentation reveals that most studies have used axial or sagittal MR images with traditional image processing techniques or CNN-based deep learning methods. However, few studies have developed a versatile segmentation application specifically for this problem using the Swin-Unet architecture, which directly integrates transformer-based attention mechanisms into the segmentation process. This study presents an innovative approach to cervical spinal cord segmentation using the Swin-Unet architecture, which utilizes multi-scale feature extraction and local and global context interaction.

As a result of the experimental studies carried out in this study, it was observed that the SWU1 model, especially the SWU1 model with swin transformer blocks integrated into the encoder part, achieved success in segmenting the cervical spinal cord with a DSC of 0.9526. This success is notable compared to classical TransUNet architectures and TransUNet variants with modern convolutional backbones. Within the study’s dataset, the DSC score of SWU1 was as high as 0.9560 on a limited raw dataset without data augmentation. Meanwhile, SWU2 and SWU3 achieved scores of 0.8945 and 0.9351, respectively. These results demonstrate that the Swin architecture achieves high accuracy through both its depth and the effective use of attentional mechanisms in the segmentation context. When evaluating TransUNet models, it is generally correct to analyze them based on backbone structures, such as classical, deep, and modern convolutions. While models with fewer layers significantly improve with increased data (TU2: +0.0045), some complex, deeper models are more sensitive to data augmentation, resulting in decreased performance (TU10: −0.0097). This difference is an important experimental outcome showing the sensitivity of model architecture to not only data size but also data variety and augmentation method.

All models were trained with the same hyperparameters during the training process (batch size = 8, learning rate = 0.001, ComboLoss = BCE + Dice, and number of epochs = 100), which allowed for fair and consistent comparisons between different architectures. Both pixel-based DSC, PRE, and REC metrics and volumetric VOE and distance-based HD95 metrics were used in model evaluations. This allows for a comprehensive analysis of the models’ segmentation performance end-to-end, considering not only local accuracy but also contour accuracy and volumetric completeness.

**Figure 6:**
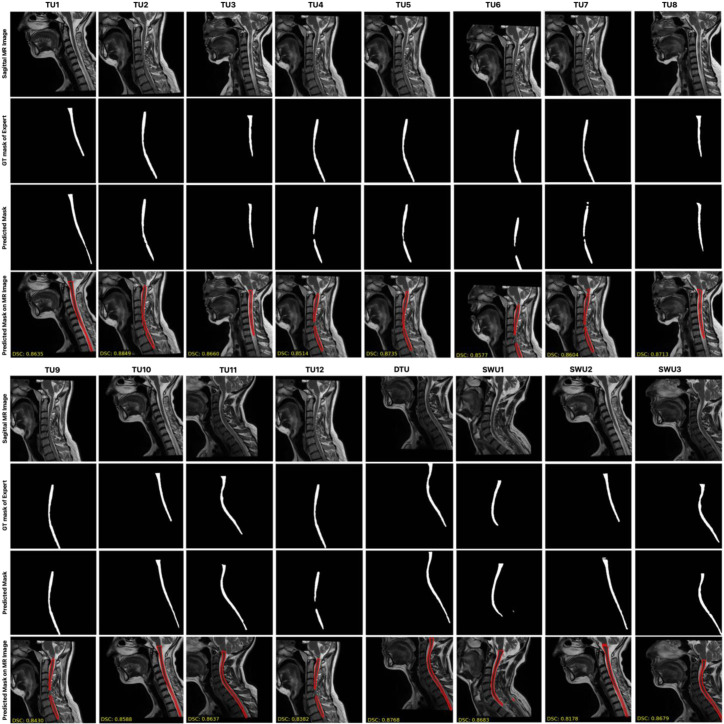
Sagittal MR images segmented by transformer-based architectures with relatively low performance for fully automated segmentation of the cervical spinal cord.

Visual analyses of the test set revealed that Swin-Unet architectures produced more consistent and anatomically appropriate segmentations. The more precise discrimination of finely structured spinal contours and poorly contrasted regions, in particular, can be attributed to the attentional mechanisms’ capacity to generate contextual awareness. With the encoder integration of Swin-based models, segmentation boundaries were better defined by examining local details in a global context. Conversely, as illustrated in Figure 6, segmentation errors in certain images resulted from limited contextual learning, inadequate model depth, or parametric overloading. Data-driven errors result from low-quality images, limited sample diversity, and inconsistencies in expert labeling. In particular, the TU1 model was able to produce accurate segmentation estimates in sagittal slices where the spinal cord structure loses clarity with image depth by exploiting contextual continuity in these regions, despite the absence of labeling. This clearly demonstrates the model’s capacity to learn context through structural continuity and attentional mechanisms. In conclusion, this study shows that, even with limited data, attention-based architectures can outperform classical CNN architectures in fully automated segmentation of the cervical spinal cord. Furthermore, the study reveals that the Swin-Unet architecture is a powerful alternative for anatomically complex structures, such as the cervical spinal cord. In light of these findings, utilizing attentional mechanisms more in-depth in combination with more balanced and diverse datasets may further improve segmentation performance.

**Figure 7:**
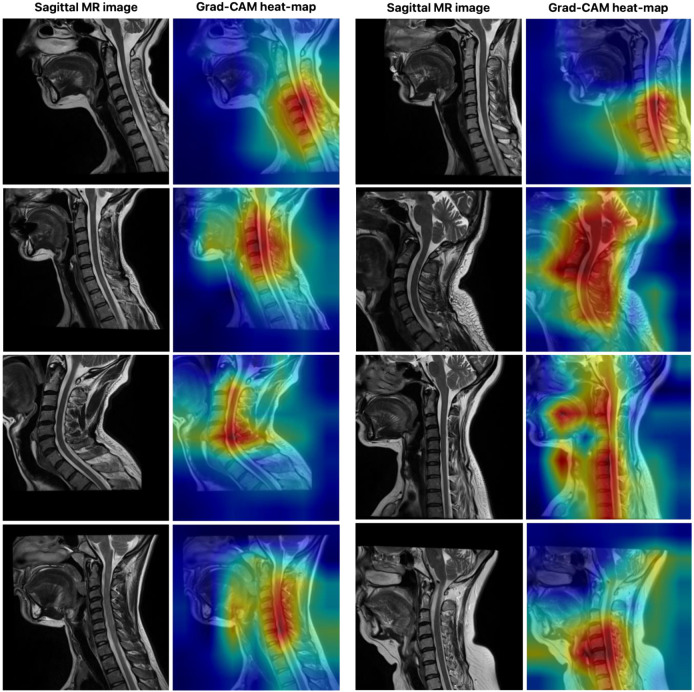
The visual explanation of the SWU1 model’s decision-making process using Grad-CAM.

Figure 7 illustrates the visual explanation of the SWU1 model’s decision-making process using Gradient-weighted Class Activation Mapping (Grad-CAM), a commonly adopted interpretability technique for convolutional and transformer-based deep learning models. The left column in each pair shows the original sagittal MR image of the cervical spine, while the right column displays the corresponding Grad-CAM heat map overlaid on the image to highlight regions of the model’s attention during segmentation. In this study, Grad-CAM was applied to the SWU1 model, which integrates swin transformer blocks in the encoder and achieved the highest segmentation performance among all evaluated models. The goal of this visualization is to reveal the spatial focus of the network-that is, which areas of the input the model deems most relevant when delineating the cervical spinal cord. The red and yellow regions on the heat maps represent higher activation zones, indicating where the model is placing the most attention during feature extraction and decision-making. As observed from the visualizations, SWU1 consistently focuses on the central spinal cord region, even in cases with anatomical curvature, varying contrast, or adjacent vertebral structures. This suggests that the attention mechanisms within the swin transformer encoder are effectively learning to prioritize semantically meaningful regions despite the presence of surrounding complex anatomy and imaging noise. Furthermore, the attention maps demonstrate spatial consistency and anatomical relevance, validating that the model does not rely on irrelevant or artifact-prone areas.

The use of Grad-CAM not only enhances interpretability and transparency of the model’s predictions-important for clinical trust and deployment-but also provides evidence of the internal feature learning dynamics of transformer-based architectures in medical image segmentation. These results reinforce the efficacy of using swin transformer blocks for high-fidelity, anatomy-aware feature representation in automated spinal cord segmentation tasks.

**Figure 8:**
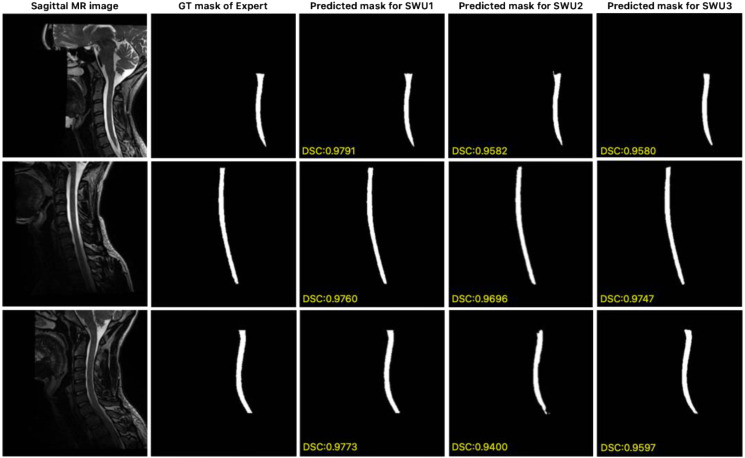
Comparison of expert GT and predicted segmentation masks generated by the proposed SWU1, SWU2, and SWU3 models on sagittal MR images of publicly available dataset.

In this study, the publicly available dataset [56] proposed by Bédard and Cohen-Adad, which consists of isotropic T2-w 3D volumes, was used to evaluate the generalization ability of the proposed SWU1, SWU2, and SWU3 models for the automatic segmentation of the cervical spinal cord. For our experiments, sagittal planes were extracted from these isotropic 3D volumes and we conducted all experiments using the full 3D volumetric data to ensure methodological consistency. The results of the key metrics obtained from the experimental studies conducted to evaluate the performance of the SWU1, SWU2, and SWU3 models are shown in Table 3. Of the three variants, SWU1 had the highest accuracy, with a DSC of 0.9540, a PRE of 0.9497, and a REC of 0.9596. It also had the lowest error rates in distance-based (HD95 = 0.5627 mm) and volume-based (VOE = 0.0868) metrics. These results confirm the stability and robustness of SWU1 in delineating cervical spinal cord regions even under anatomical and contrast variability. In contrast, although SWU2 and SWU3 produced competitive PRE values (0.9253 and 0.9329, respectively), they showed lower DSC and REC scores and relatively higher HD95 and VOE values. This indicates weaker boundary consistency and spatial localization. Overall, the findings suggest that the encoder-enhanced SWU1 architecture effectively balances local detail preservation and global context modeling. This makes it the most clinically promising framework among the evaluated models. In Figure 8, segmentation results from the publicly available dataset are presented, comparing the expert-generated GT masks with the predicted masks obtained by the proposed SWU1, SWU2, and SWU3 models. The visual analysis reveals that the SWU1 model achieves highly accurate segmentation, closely matching the expert annotations, particularly in thin and curved regions of the cervical spinal cord, with DSC scores.

The SWU1, SWU2 and SWU3 models, which are based on Swin-Unet and were proposed in this study for automatically segmenting T2-w sagittal MR images of the cervical spinal cord, were compared with the results of some state-of-the-art methods. Table 4 shows the segmentation performance of the SWU1, SWU2 and SWU3 models compared with several state-of-the-art methods on the test set of the dataset. The PropSeg (De Leener et al. [29]), sc_contrast_agnostic (Bédard et al. [36]), DeepSeg2D (Gros et al. [30]), and DeepSeg3D (Gros et al. [30]) methods were run on the Spinal Cord Toolbox framework. On the other hand, nnU-Net (Isensee et al. [57], Isensee et al. [58]) achieved segmentation performance results using its own framework [59]. The classical PropSeg method achieved the lowest DSC of 0.7676, confirming its limited ability to capture fine structural details of the cervical spinal cord. In addition, the state-of-the-art method sc_contrast_agnostic, a deep learning-based method, resulted in a DSC score of 0.8906. In contrast, the other deep learning-based methods, including DeepSeg3D, nnU-Net and DeepSeg2D produced substantially better results with DSC scores ranging from 0.9309 to 0.9331. However, the proposed Swin-Unet variants SWU1, SWU2 and SWU3 outperformed these methods. SWU2 and SWU3 achieved DSC scores of 0.9365 and 0.9456, respectively. On the other hand, the SWU1 architecture outperformed all methods, achieving a DSC of 0.9526. These results illustrate the effectiveness of integrating swin transformer blocks into the segmentation pipeline, enabling the models to jointly capture global contextual dependencies and local anatomical details. The improvement over other transformer and CNN-based architectures underscore the Swin-Unet framework’s robustness and precision for challenging sagittal MR views. These results suggest that the proposed models could be valuable tools for automated cervical spinal cord analysis, supporting radiological workflows and clinical decision-making.

Accurate segmentation of the cervical spinal cord is essential for diagnosing and monitoring various neurological disorders. Conditions such as MS and traumatic spinal cord injuries often present with subtle or overlapping symptoms. Early and precise identification of these conditions can significantly impact treatment outcomes. For example, in MS, monitoring spinal cord atrophy through accurate segmentation aids in assessing disease progression and treatment efficacy. The proposed Swin-Unet models demonstrated high segmentation accuracy using the SWU1 model on sagittal T2-w MR images. This performance surpasses existing state-of-the-art methods, highlighting the effectiveness of integrating swin transformer blocks in the encoder to capture local and global contextual information. The SWU1 model’s high accuracy and interpretability have significant clinical implications. Automated and precise segmentation can help clinicians identify pathologies, such as spinal cord compression or atrophy, and facilitate timely intervention. Additionally, quantitative measurements derived from accurate segmentation can serve as biomarkers for assessing disease progression in conditions like MS.

Integrating the proposed segmentation framework into radiology workflows has the potential to significantly improve diagnostic efficiency and accuracy. Current clinical practice involves time-consuming manual delineation of the cervical spinal cord, which is prone to inter-observer variability, especially in cases with low contrast or structurally complex regions. The proposed method could be embedded into radiology software platforms or PACS systems to assist radiologists during routine evaluations by providing fully automated, highly accurate segmentation within seconds. For instance, automated segmentation masks could be generated immediately after MR acquisition, allowing radiologists to review the results alongside the original images without additional manual effort. This could accelerate the diagnosis of cervical pathologies, such as multiple sclerosis, spinal cord tumors, and degenerative diseases, by enabling precise volumetric and morphometric analyses. Furthermore, automated segmentation could facilitate longitudinal monitoring, in which consistent delineation across time points is essential for tracking disease progression or treatment response. It is important to note that such integration would not replace radiologists, but rather serve as a decision support tool. It would improve reliability, reduce workload, and ultimately enhance patient care. From a clinical perspective, the computational efficiency of the proposed Swin-Unet variants is important for their real-world deployment in radiology workflows. With nearly 99 million trainable parameters each, SWU1 and SWU3 demonstrated high computational demands yet maintained consistent GPU memory usage of around 397–399 MB. This makes them suitable for high-performance clinical workstations. SWU2, with only 7.7 million parameters, is a more lightweight alternative that consumes substantially less memory (170 MB). This makes it suitable for deployment on less resource-intensive systems, such as portable diagnostic devices. However, its longer training duration (105 min) compared to SWU1 (89 min) suggests potential optimization limitations that could affect scalability. These results suggest that, clinically, SWU1 provides a balanced trade-off between model complexity and efficiency, making it a robust option for integration into routine hospital imaging systems. In contrast, SWU2 could be more advantageous in resource-constrained environments where rapid deployment and reduced computational costs are prioritized.

The proposed frameworks based on Swin-Unet demonstrated promising segmentation performance on sagittal cervical spinal cord MR images, highlighting its potential for clinical use in reducing manual workload and inter-observer variability. However, there are some limitations in terms of the generalizability of the results to wider clinical applications. First, although the results demonstrate strong segmentation accuracy, the framework’s clinical applicability remains to be validated. The study is limited by its reliance on publicly available datasets with controlled imaging protocols, which may not capture the variability of routine clinical practice. In addition, the dataset used was privately created and is not publicly available. This restricts direct comparisons with similar approaches and the external validity of the results. A significant barrier to the wider dissemination of model development in this field is the lack of open-access, well-annotated, and multicenter datasets for cervical spinal cord segmentation. Furthermore, although the dataset used in this study consists of sagittal T2-w MR images of 98 patients and was increased to 869 samples by data augmentation methods, the limited anatomical diversity of these images may limit the generalization capacity of the model. Another factor affecting segmentation accuracy is that some MR images in the dataset have unclear spinal cord contours due to low contrast and poor structural visibility. In these regions, annotation and structural awareness can be challenging. The ability of models to make successful predictions in these areas thanks to contextual learning demonstrates the capacity of these types of architectures to predict based on structural continuity. However, it is important to re-evaluate expert annotations in cases where segmentation errors are observed to improve the quality of the training process and the accuracy of the model outputs.

## 5. Conclusions

In this study, we applied transformer-based Swin-UNet architectures for automated cervical spinal cord segmentation using sagittal MR images. The SWU1 model achieved the highest performance with 0.9526 DSC score, surpassing convolution-based and TransUNet variants with swin transformer blocks in the encoder. The SWU1 model’s success stems from its integration of attention mechanisms that capture both local details and global context, ensuring stable segmentation in low-contrast and structurally variable regions. Multidimensional evaluations with the key metrics such as DSC, PRE, REC, VOE, and HD95 confirmed robust accuracy, and Grad-CAM visualizations demonstrated anatomically meaningful attention. Despite being trained on a limited, augmented dataset, the models generalized effectively, supporting clinical applications in monitoring MS, spinal tumors, and degenerative diseases. However, limitations include dataset size, anatomical diversity, and variability in image quality. Future work could involve larger multicenter datasets, model optimization, and systematic error analysis, and include pilot testing in radiology departments using prospective, heterogeneous patient data to evaluate the method’s robustness, workflow integration, and diagnostic efficiency. Overall, Swin-Unet provides a precise, clinically promising framework for cervical spinal cord segmentation.

## Figures and Tables

**Table 1:** Transformer-based segmentation models used in this study and their properties.

Model	Transformer Variant	Critical Features
TU1	TransUNet (ResNet-34)	Lightweight construction, low computational cost
TU2	TransUNet (ResNet-50)	Deep structure, bottleneck blocks
TU3	TransUNet (ResNet-101)	Increased depth, more capacity
TU4	TransUNet (ResNet-152)	Very deep structure, high accuracy
TU5	TransUNet (ResNet-50d)	Large kernel structure, optimized version
TU6	TransUNet (ResNet-101d)	Deep and advanced ResNet variant
TU7	TransUNet (ResNeXt50_32×4d)	Grouped convolutions, efficient parameter usage
TU8	TransUNet (ResNeSt50d)	Better multi-scale learning with Split-Attention module
TU9	TransUNet (ResNetV2_50×1)	Pre-activation ResNet, BiT (Big Transfer) model
TU10	TransUNet (EffNetV2-s)	MobilNet-style structure, fast and efficient
TU11	TransUNet (ConvNeXt-tiny)	CNN and Transformer combined
TU12	TransUNet (RegNetY_040)	Automatically designed CNN, parametric efficiency
DTU	TransUNet (Deco-TransUNet)	Transformer-based decoder UNet structure
SWU1	Swin-Unet (Encoder Swin Transformer)	Window-based attention, high-resolution segmentation
SWU2	Swin-Unet (Decoder Swin Transformer)	Modeling semantic details, especially in the upsampling phase
SWU3	Swin-Unet (Encoder–Decoder Swin Transformer)	effectively represent both global context and local details

**Table 2:** Quantitative evaluation of the proposed Swin-Unet-based models alongside pretrained TransUNet variants for segmentation of the cervical spinal cord from sagittal MR images.

Architecture	DSC	PRE	REC	HD95 [mm]	VOE
TU1	0.9406 ± 0.02	0.9411 ± 0.03	0.9415 ± 0.03	1.1684 ± 0.19	0.1112 ± 0.03
TU2	0.9409 ± 0.02	0.9380 ± 0.03	0.9450 ± 0.02	1.2225 ± 0.21	0.1108 ± 0.03
TU3	0.9408 ± 0.02	0.9418 ± 0.03	0.9412 ± 0.03	0.9306 ± 0.12	0.1109 ± 0.03
TU4	0.9391 ± 0.02	0.9443 ± 0.03	0.9354 ± 0.03	1.1852 ± 0.20	0.1139 ± 0.03
TU5	0.9382 ± 0.02	0.9280 ± 0.03	0.9503 ± 0.03	1.6308 ± 0.27	0.1154 ± 0.04
TU6	0.9396 ± 0.02	0.9446 ± 0.03	0.9363 ± 0.03	1.2850 ± 0.20	0.1131 ± 0.03
TU7	0.9382 ± 0.02	0.9260 ± 0.03	0.9521 ± 0.03	1.3206 ± 0.22	0.1156 ± 0.04
TU8	0.9360 ± 0.02	0.9107 ± 0.03	0.9643 ± 0.02	2.4751 ± 0.31	0.1193 ± 0.04
TU9	0.9401 ± 0.02	0.9418 ± 0.03	0.9400 ± 0.03	1.3258 ± 0.24	0.1121 ± 0.04
TU10	0.9424 ± 0.02	0.9392 ± 0.03	0.9468 ± 0.02	1.1788 ± 0.22	0.1081 ± 0.03
TU11	0.9436 ± 0.02	0.9334 ± 0.03	0.9549 ± 0.02	1.0229 ± 0.16	0.1059 ± 0.04
TU12	0.9415 ± 0.02	0.9396 ± 0.03	0.9450 ± 0.03	1.2771 ± 0.23	0.1096 ± 0.04
DTU	0.9413 ± 0.02	0.9350 ± 0.03	0.9489 ± 0.02	1.1663 ± 0.21	0.1101 ± 0.03
SWU1	0.9526 ± 0.02	0.9477 ± 0.03	0.9587 ± 0.02	1.0707 ± 0.18	0.0896 ± 0.03
SWU2	0.9365 ± 0.02	0.9287 ± 0.04	0.9467 ± 0.03	1.6959 ± 0.18	0.1183 ± 0.04
SWU3	0.9456 ± 0.02	0.9473 ± 0.03	0.9458 ± 0.03	1.1235 ± 0.13	0.1022 ± 0.04

**Table 3:** Performance evaluation of SWU1, SWU2, and SWU3 architectures on the publicly available sagittal T2-w cervical spinal cord MR dataset.

Proposed Architectures	DSC	PRE	REC	HD95 [mm]	VOE
SWU1	0.9540 ± 0.02	0.9497 ± 0.04	0.9596 ± 0.02	0.5627 ± 0.11	0.0868 ± 0.04
SWU2	0.9144 ± 0.04	0.9253 ± 0.07	0.9117 ± 0.06	1.3614 ± 0.14	0.1543 ± 0.07
SWU3	0.9179 ± 0.04	0.9329 ± 0.06	0.9099 ± 0.06	1.1991 ± 0.13	0.1488 ± 0.07

**Table 4:** Performance comparison of the proposed SWU1 model with state-of-the-art methods for cervical spinal cord segmentation on sagittal T2-w MR images based on DSC metric.

Methods	DSC
PropSeg (De Leener et al. [29])	0.7676 ± 0.13
sc_contrast_agnostic (Bédard et al. [36])	0.8906 ± 0.05
DeepSeg3D (Gros et al. [30])	0.9309 ± 0.05
nnU-Net (Isensee et al. [57])	0.9326 ± 0.10
DeepSeg2D (Gros et al. [30])	0.9331 ± 0.04
Ours (Swin-Unet-based SWU2)	0.9365 ± 0.02
Ours (Swin-Unet-based SWU3)	0.9456 ± 0.02
Ours (Swin-Unet-based SWU1)	0.9526 ± 0.02

## Data Availability

Dataset available on request from the authors.
